# CD14^lo^CD301b^+^ macrophages gathering as a proangiogenic marker in adipose tissues

**DOI:** 10.1016/j.jlr.2024.100720

**Published:** 2024-12-05

**Authors:** Yibing Lv, Yidan Zheng, Shanshan Su, Junyi Xiao, Jie Yang, Lingyun Xiong, Yanyan Guo, Xiaoqi Zhou, Nengqiang Guo, Ping Lei

**Affiliations:** 1 Department of Immunology, School of Basic Medicine, Tongji Medical College, Huazhong University of Science and Technology, Wuhan, China; 2 Henan Provincial Key Laboratory of Genetic Diseases and Functional Genomics, Medical Genetic Institute of Henan Province, Henan Provincial People’s Hospital, People’s Hospital of Zhengzhou University, Zhengzhou, China; 3 Department of Plastic Surgery, Union Hospital, Tongji Medical College, Huazhong University of Science and Technology, Wuhan, China; 4 Department of Transfusion Medicine, Wuhan Hospital of Traditional Chinese and Western Medicine, Tongji Medical College, Huazhong University of Science and Technology, Wuhan, China

**Keywords:** angiogenesis, CD14, CD301b, obesity, macrophages

## Abstract

The role of the monocyte marker CD14 in the regulation of obesity is increasingly recognized. Our observations indicated that *Cd14*^−/−^ mice exhibited a leaner body shape compared to their wild-type (WT) counterparts. And the loss of CD14 alleviated high-fat diet–induced obesity in mice. In human subjects, CD14 level was tested to be positively correlated with overweight and obesity. However, the relationship between CD14 and the development of obesity remains only partially understood. To investigate the underlying mechanisms, adipose tissues (ATs) from *Cd14*^−/−^ and WT mice were subjected to deep RNA sequencing. Gene Ontology enrichment analysis revealed a significant enhancement of angiogenesis-related function in the *Cd14*^−/−^ epididymal adipose tissues compared to WT counterpart, which was accompanied by an upregulation of *Cd301b*. Subsequent assays confirmed the enhanced angiogenesis and more accumulation of CD301b^+^ macrophages in *Cd14*^−/−^ epididymal adipose tissues. Because *Igf1* expression has been suggested to be associated with *Cd301b* expression through pseudotime analysis, we found it was insulin-like growth factor 1 secreted from *Cd14*^−/−^ macrophages that mediated the angiogenesis enhancement. Collectively, our ﬁndings indicate that CD14 deficiency increased the accumulation of CD14^lo^CD301b^+^ macrophages in ATs, which may serve as a proangiogenic marker, providing novel insights into the relationship between CD14 and obesity development.

CD14 is a well-recognized pattern-recognition receptor that serves as a coreceptor for Toll-like receptor 4, playing a critical role in innate immune responses. It recognizes bacterial lipopolysaccharides as well as pathogen- and damage-associated molecular patterns to facilitate inflammatory immune responses (1). Additionally, CD14 binds oxidized lipids to initiate noninfectious inflammatory responses (2). Beyond its role in immune responses, CD14 has been recognized for its broader role in regulating metabolism, insulin resistance, and obesity (3, 4). For instance, in obese individuals, CD14 expression is elevated in adipose tissues (ATs) and shows a positive correlation with the development of obesity (5, 6). CD14-deﬁcient mice exhibit an ideal body composition with reduced body fat and are resistant to high-fat diet (HFD)–induced obesity, insulin resistance, and dyslipidemia, compared to their wild-type (WT) counterparts (7).

Although the connection between CD14 and metabolic regulation is partially understood, it has been observed that obese WT mice transplanted with *Cd14*^−/−^ bone marrow exhibit resistance to the development of insulin resistance (6). This finding suggests that CD14-expressing immune cells may exacerbate obesity development. CD14 is recognized as a cell surface marker on monocytes and macrophages (8). Macrophages constitute the largest proportion of immune cells in AT and play a ubiquitous role in adipose immunology (9). These macrophages can be further classified into various unique populations, each with distinct functions. For example, sympathetic neuron-associated macrophages modulate adipocyte metabolism and adaptive thermogenesis by influencing adrenergic signaling and sympathetic innervation/tone within the tissue (10). Lipid-associated macrophages facilitate phagocytosis, lipid catabolism, and metabolic reprogramming in a TREM2-dependent manner during obesity (11). Metabolically activated macrophages primarily sequester lipids through the formation of lysosomal synapses with dead or dying adipocytes, thereby preventing lipotoxicity from necrotic adipocytes and resulting in their proinflammatory activation (12). A distinct phagocytic macrophage population also accumulates in response to caloric restriction (13). Additionally, LYVE-1-positive macrophages promote angiogenesis in AT to prevent hypoxia and facilitate metabolic communication between adipocytes and the circulation (14). These adipose tissue macrophages (ATMs) play a crucial role in regulating inflammation and metabolic homeostasis, orchestrating tissue expansion, innervation, thermogenesis, and influencing the progression of diseases, such as cancer or diabetes (15, 16).

To investigate the linked mechanisms by which CD14-expressing immune cells regulate the development of obesity, this research analyzed gene differences between *Cd14*^−/−^ and WT ATs, based on the observation that *Cd14*^−/−^ mice exhibited an ideal body composition with reduced body fat compared to their WT counterparts. Gene set enrichment (GSE) analysis and subsequent experiments revealed that *Cd14*^−/−^ ATs were enriched with a high accumulation of CD301b^+^ macrophages and increased level of insulin-like growth factor 1 (IGF-1), thereby enhancing the angiogenic potential of the AT. Our findings suggest an angiogenic role for *Cd14*^−/−^ macrophages in epididymal AT, providing novel insight into the complex interplay between CD14 and the development of obesity.

## Materials and Methods

### Animals

C57BL/6J mice (HFK Bioscience, Beijing, China) and C57BL/6J background *Cd14*^−/−^ mice (The Jackson Laboratory, Bar Harbor, ME), aged 6–8 weeks, were fed either a normal chow diet (12% kcal from fat; HFK Bioscience) or a HFD (60% kcal from fat; MD12033, Medicine, China) for a duration of 16 weeks. All mice had unrestricted access to food and water. Body weights were recorded weekly. Following the euthanization of the mice, epididymal visceral AT was harvested and weighed for subsequent analysis. All animal experiments were approved by the Animal Ethical Committee of Tongji Medical College, Huazhong University of Science and Technology (Approval ID: S1161/S2534).

### RNA sequencing and bioinformatic analysis

mRNA extracts from cells and tissues were subjected to deep RNA sequencing (RNA-seq) analysis (SEQHEALTH, Wuhan, China). The prepared mRNA libraries were sequenced on an Illumina HISEQ 2500, and the Hisat2 v2.0.4 software (the Center for Computational Biology, Baltimore, MD) was used to map cleaned reads to the mm10 reference genome. Fragments per kilobase of exon per million mapped fragments were calculated using Cuffnorm, version 2.2.1 software (University of Washington, Seattle, WA). Genes with a normalized fragments per kilobase of exon per million mapped fragments value > 1.0 were identified. Differentially expressed genes were defined statistically significant as having a log2 (fold change) ≥ 0.5 and a *P* value ≤ 0.05. Heatmaps and volcano plots were generated using the R package.

The scRNA-seq database (GSE176067) was utilized to analyze monocyte/macrophage populations in ATs. Gene ontology (GO) analysis was performed using the clusterProfiler R package (https://github.com/YuLab-SMU/clusterProfiler), which constructed GO annotations for biological process. Pseudotime analysis was conducted using the R package Monocle 3 (https://cole-trapnell-lab.github.io/monocle3), allowing for the detection of differentiation pathways and the establishment of gene expression levels across pseudotime for CD14^low^ cells.

### Human samples

Adult human subcutaneous white adipose tissues (scWATs) were obtained from individuals undergoing abdominoplasty in Wuhan Union Hospital (Tongji Medical College, Huazhong University of Science and Technology). Half of the fresh scWATs were minced for cell isolation, while the remainder was used for immunofluorescence staining. Participants were categorized into 2 groups: overweight/obese [body mass index (BMI)＞25 kg/m^2^, n = 4] and healthy weight (BMI＜25 kg/m^2^, n = 4) groups. All individuals were free from any evident systemic diseases and chronic infections. Informed consent was obtained from all subjects. Experiments involving human participants were approved by the Ethical Committee of Tongji Medical College, Huazhong University of Science and Technology (Approval ID: S069). Furthermore, all procedures involving human subjects adhered to the principles outlined in the Declaration of Helsinki.

### Cell isolation

Bone marrow–derived macrophages (BMDMs) were induced following the protocol outlined in our previous report (17). In brief, the tibiae and femurs of mice were excised, and the bone marrow cells were flushed out. Following a red blood cell lysis procedure, these cells were cultured in RPMI 1640 medium supplemented with recombinant mouse M-CSF (20 ng/ml, 576408, Biolegend, San Diego, CA) for a duration of 7 days. Subsequently, the cells were further stimulated with rmIL-4 (20 ng/ml, 574308, Biolegend) or lipopolysaccharide (LPS) (100 ng/ml, 0111:B4, L3024, Sigma-Aldrich) for 36 h, after which the cell pellet and supernatant were collected for subsequent analyses.

The stromal vascular fraction cells (SVFs) and ATMs were obtained according to previously established methodologies (18). Briefly, AT samples were enzymatically digested in 0.1% collagenase Ⅱ (LS004176, Worthington, Lakewood, NJ) in Hank’s buffer at 37 °C for 45 min. SVFs were collected by centrifugation at 1,500 rpm for 10 min, after which the cells were washed 3 times with PBS and filtered through a 70 μm cell strainer. Red blood cells were lysed using ACK lysis buffer (G2015, Servicebio, Wuhan, China). The cells were then resuspended in 1 ml PBS for further analysis. To isolate ATMs, F4/80 positive beads (100-0659, StemCell Technologies, Vancouver, BC, Canada) were utilized to purify macrophage from the SVFs. All procedures adhered to the manufacturer's instructions.

### Histological and morphological analysis

ATs were fixed in 4% paraformaldehyde for 24 h, embedded in paraffin, and then sectioned into 4-μm thickness. H&E staining was performed as described in our previous report (17). For immunohistochemical staining, the sections were treated with a citric acid solution (pH 6.0, 95–100 °C) to retrieve the antigens, followed by incubation with 3% H_2_O_2_ for 15 min, and 5% serum for 1 h. The sections were then probed overnight with αCD31 (ab182981, Abcam, Cambridge, MA) and αENDO (A8494, ABclonal, Wuhan, China), developed using VECTORSTAIN ABC kit (PK4001, Vector Laboratories Inc., Burlingame, CA), and further counterstained with Harris’ hematoxylin. For immunofluorescence staining, sections were incubated with αCD31, αCD301b (14-3011-82, eBioscience, San Diego, CA), αCLEC10A (354703, BioLegend), αCD68 (A22329, ABclonal, Wuhan, China), and αF4/80 (70076, CST, Danvers, MA), followed by incubation with Alexa Fluor-conjugated secondary antibodies. DAPI (D1306, Invitrogen, Carlsbad, CA) was used to stain the nuclei. Immunofluorescence signals were visualized using a fluorescence microscope (Pannoramic MIDI, 3DHISTECH, Hungary). The mean lipid droplet surface area in H&E-stained adipose sections was quantified in a blinded manner using Image-Pro Plus software (Version X; Media Cybernetics, Silver Springs, MD).

### Real-time quantitative PCR

Total RNA was extracted from both cells and ATs, and cDNA was synthesized as described in our previous report (17). The SYBR Green Real-Time PCR kit (1725124, Bio-Rad, Hercules, CA) was employed to prepare the RT-PCR mixture. β-Actin was used to normalize the mRNA levels, and fold changes were calculated using the 2^−△△Ct^ method.

### ELISA

The concentration of IGF-1 was measured using an ELISA kit (E-EL-M3006, Elabscience Biotechnology Co.) according to the manufacturer’s recommendations. Briefly, 100 μl samples were incubated in a precoated plate for 90 min. Then, samples were coated with a biotinylated detection antibody for 1h, followed by incubated with HRP conjugate for 30 min, and finally with substrate reagent for 15 min. The absorbance was measured at 450 nm, and the IGF-1 concentration was calculated in accordance with the manufacturer's guideline.

### Proliferation assay and tube formation

The endothelial cell line bEND.3 was mixed with the culture supernatant of BMDMs or SVFs isolated from ATs for 48 h. The CCK8 kit (BS350 B, Biosharp, China) was utilized to assess cell proliferation. Tube formation assay was performed by seeding bEND.3 cells (30,000 cells/well) in a 96-well plate precoated with a growth factor-reduced basement membrane matrix (082724, ABW, Shanghai, China) in the presence of DMEM or harvested conditioned media. Four hours later, images were captured using a Nikon light microscope (Japan) under bright field conditions to document the cells and developed tubules. Angiogenesis-related total length was analyzed using ImageJ software with the Angiogenesis Analyzer plugin (NIH, Bethesda, MD).

### Flow cytometry

The detailed methods for cell staining were described in our previous reports (19). In summary, cells were blocked with CD16/CD32 antibodies (101320, Biolegend) and then stained with the following antibodies for 30 min on ice, including CD45 (101038, Biolegend), F4/80 (123110, Biolegend), CD11b (101226, Biolegend), CD301b (146814, Biolegend), CD206 (141704, Biolegend), and CD11c (558079, BD) antibodies for mice SVFs. For human SVFs, the staining included CD45 (555482, BD), CD68 (564943, BD), CD11b (301310, Biolegend), CD301/CLEC10a (354704, Biolegend), and CD14 (325618, Biolegend) antibodies. Flow cytometry data were acquired using FASC verse (BD Biosciences, San Jose, CA) and analyzed with FlowJo software (v10.5.3, Flow Jo LLC, Ashland, OR).

### Metabolic-related index detection

The Comprehensive Laboratory Animal Monitoring System (Columbus Instruments) was utilized to measure energy expenditure (EE), food intake, O_2_ consumption, and activity in both WT and *Cd14*^−/−^ mice. Prior to data collection, all mice were individually housed and adapted to the metabolic cages for 48 h. During the monitoring period, each mouse had unrestricted access to food and water, and all relevant data were collected using the sensors of this system. Calr2 (https://CalRapp.org) was employed to visualize the collected data on EE and food intake of mice, as previous report (20). The EE of the 2 groups of mice was estimated using ANCOVA, incorporating body weight as a covariate in the analysis. This approach examined whether the effect of the body mass covariate on EE was consistent between WT and *Cd14*^−/−^ mice, thereby highlighting the role of CD14 deficiency in EE (20, 21).

### Statistical analysis

Statistical comparisons were conducted using GraphPad Prism 7 (GraphPad Software Inc., San Diego, CA). An unpaired Student's *t* test was employed to compare 2 groups, while one-way analysis of variance (ANOVA) was utilized for comparisons among multiple groups. Images were generated using GraphPad Prism 7. (GraphPad Software Inc., San Diego).

## Results

### Loss of CD14 alleviates HFD-induced obesity with enhanced adipose angiogenesis in mice

**Fig. 1:**
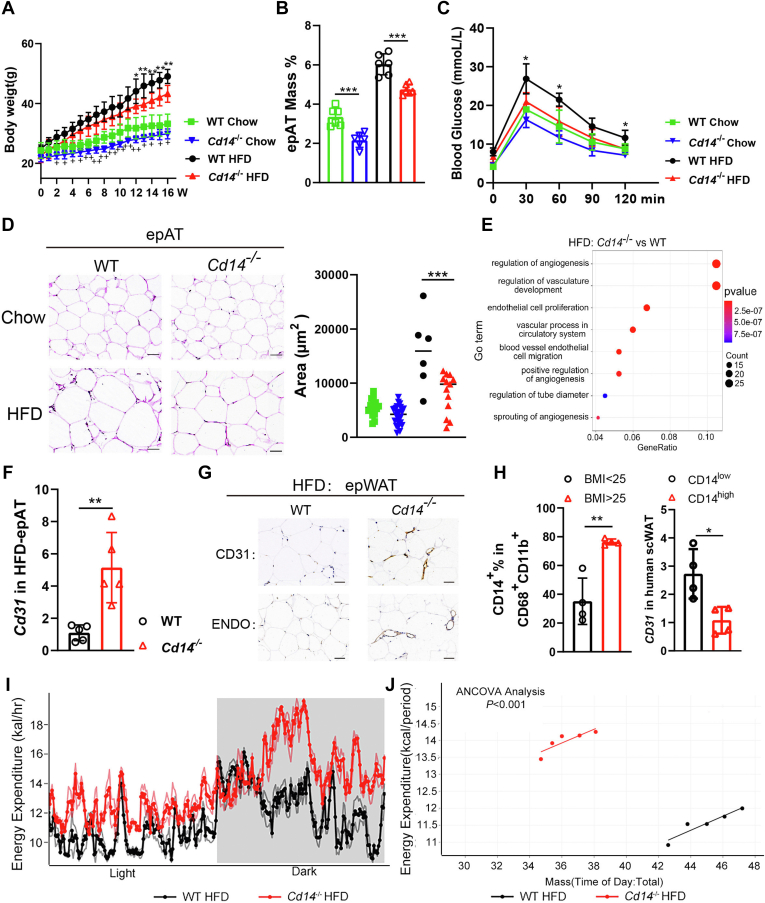
CD14 deletion alleviates HFD-induced obesity with more angiogenesis in adipose tissues. (A) Body weight and (B) epWAT mass of HFD- or chow-fed WT and *Cd14*^*−/−*^ mice. (C) GTT analysis for WT and *Cd14*^*−/−*^ mice, and the significant difference was shown between HFD-induced WT and *Cd14*^*−/−*^ mice. (D) Representative H&E staining (left) and adipocytes size quantiﬁcation (right) for epWAT (scare bar: 50 μm). (E) GO functional enrichment analysis for epWAT. The color of each bubble represents the *P* value, and bubble size represents the gene number. (F) *Cd31* mRNA expression in epWATs of HFD-fed mice. (G) Immunohistochemical staining for CD31 and Endomucin in epWAT of HFD-fed mice (Scare bar: 50 μm). (H) The frequencies of CD14^+^ ATMs in overweight/obese (BMI＞25 kg/m^2^) and healthy weight (BMI＜25 kg/m^2^) individuals (left panel) and *CD31* mRNA levels in CD14^hi^ and CD14^lo^ individuals (right panel), n = 4. (I) Energy expenditure curves and (J) ANCOVA analysis in HFD-fed mice. In A: ^+^*P* < 0.05, ^++^*P* < 0.01 mean the significant difference between chow-induced WT and *Cd14*^*−/−*^ mice, and other ∗*P* < 0.05, ∗∗*P* < 0.01, ∗∗∗*P* < 0.001 mean the significant difference between groups in this figure. epWAT, epididymal adipose tissue.

To investigate the role of CD14 in the development of obesity, WT and *Cd14*^−/−^ mice were fed a HFD for 16 weeks. Notably, both HFD- and Chow-fed *Cd14*^−/−^ mice exhibited lower body weights compared with their counterparts, suggesting that CD14 deficiency mitigated obesity development and weight gain (Fig. 1A). To further explore whether CD14 deficiency inhibited the expansion of ATs, epididymal adipose tissue (epWAT) mass and the size of adipocytes were measured. It was observed that both HFD-induced and Chow-fed *Cd14*^−/−^ mice had reduced epWAT mass and smaller adipocyte sizes compared to WT mice. Mice fed with long-term HFD typically exhibited impaired glucose tolerance. Our glucose tolerance test analysis revealed that *Cd14*^−/−^ mice had lower glucose levels than WT mice at the designated timepoint following HFD induction (Fig. 1B–D). These results indicated that the absence of CD14 ameliorated the HFD-induced obesity development.

To gain insights into the mechanisms by which CD14 regulates the lipid storage and the expansion of ATs, bulk RNA-seq was performed on epWATs. GO enrichment analysis demonstrated that *Cd14*^−/−^ epWATs exhibited significant enrichment in proangiogenic functions, particularly those associated with angiogenesis and vascular development (Fig. 1E), suggesting that loss of CD14 enhanced angiogenesis during obesity development. Angiogenesis in ATs is crucial for controlling adipocyte metabolism and is essential for tissue remodeling (22, 23). Adequate oxygen and nutrients are primarily supplied by the dense vasculature in ATs, while excessive obesity impairs vascular remodeling, which can further induce metabolic disorders in ATs (24). To investigate the proangiogenic effects in *Cd14*^−/−^ mice, CD31, a well-established biomarker for angiogenesis during vascularization (25), was analyzed in epWATs. RT-qPCR results demonstrated a significant increase in *Cd31* mRNA expression in HFD-induced *Cd14*^−/−^ epWATs compared to their WT counterparts (Fig. 1F). Additionally, immunohistochemical staining revealed greater blood vessel formation in both HFD-induced (Fig. 1G) and chow-fed (supplemental Fig. S1A) *Cd14*^−/−^ epWATs than in WT tissues. These findings suggested that the angiogenic effect was independent of HFD feeding and that CD14 played negative role in adipose angiogenesis.

To further investigate the role of human CD14 in the development of obesity and adipose angiogenesis, adult human scWAT samples were collected from individuals undergoing abdominoplasty. SVFs were isolated to analyze the correlation between the frequencies of CD14^+^ macrophages and BMI. The results indicated that in individuals with a BMI greater than 25, the proportion of CD14^+^ macrophages significantly increased, rising from 35.15% ± 16.00% in the healthy weight group to 76.40% ± 2.05% in the overweight/obese group. Additionally, in individuals with high CD14 expression (CD14^high^), the *CD31* mRNA expression was markedly lower than those in the CD14 low expression group (CD14^low^) (Fig. 1H).

To investigate whether the changes observed in *Cd14*^−/−^ epWATs could influence energy metabolism in mice, EE in HFD-induced mice was measured using the Comprehensive Laboratory Animal Monitoring System. The results indicated that HFD-induced *Cd14*^−/−^ mice exhibited significantly enhanced EE (Fig. 1I), while showing comparable levels of food intake compared to WT mice (supplemental Fig. S1B). ANCOVA analysis further confirmed that EE of WT and *Cd14*^−/−^ mice increased with body weight increment in similar slopes, while *Cd14*^−/−^ mice showed significantly higher EE compared to WT mice (Fig. 1J). To further investigate whether the enhanced EE in *Cd14*^−/−^ mice were influenced by HFD feeding, chow-fed mice were also examined. As illustrated in supplemental Fig. S1B, C, chow-fed *Cd14*^−/−^ mice exhibited significantly greater EE compared to WT mice, while there was no difference in food intake between chow-fed WT and *Cd*14^−/−^ mice. This finding suggested that genetic CD14 deficiency affected EE independent of HFD feeding. Based on this result, it could be speculated that the increased EE observed in *Cd14*^−/−^ mice may contribute to their resistance to obesity and the maintenance of a lower body weight during the development of obesity induced by HFD-feeding.

Above data suggest that the loss or reduced expression of CD14 mitigates HFD-induced obesity by promoting angiogenesis and increasing EE in ATs.

### CD14 deletion upregulates the frequency of CD301b^+^ ATMs in HFD mice

**Fig. 2:**
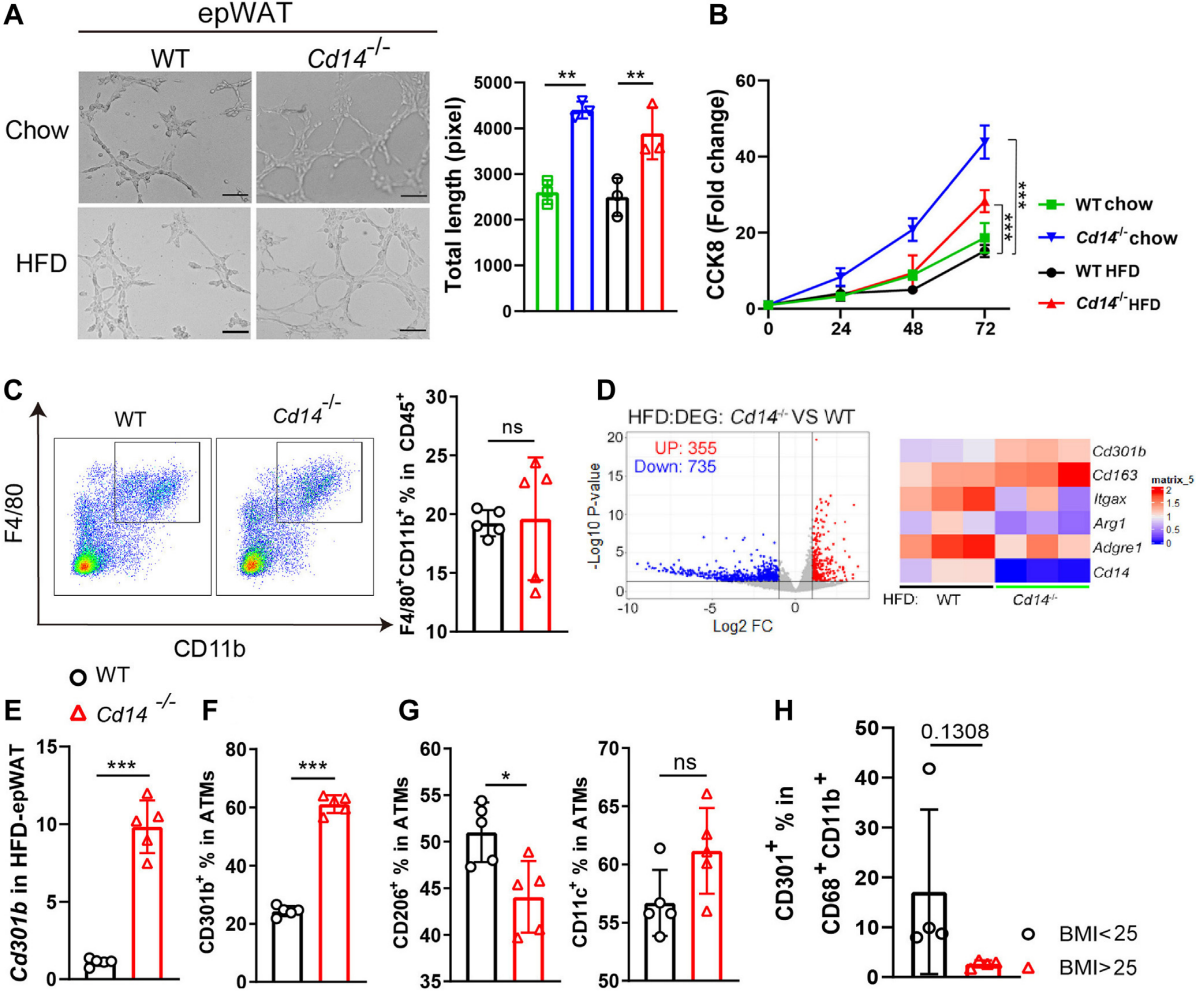
Loss of CD14 upregulates the frequency of CD301b^+^ macrophages in adipose tissue. (A) Representative images of tube formation (left) and total tube length (right) by bEND.3 cells conditioned with supernatant of epWAT-harvested SVFs. (B) Proliferation assay for bEND.3 cell. (C) The proportion of F4/80^+^CD11b^+^ macrophages in epWAT of HFD-fed mice. (D) Volcano plot and heatmap representing the differentially expressed gene level in *Cd14*^*−/−*^ relative to WT mice. The data were generated from RNA deep sequencing as described. (E) RT-qPCR analysis for the relative mRNA abundance of *CD301b* in epWAT. Frequencies of (F) CD301b^+^, (G) CD206^+^, and CD11c^+^ ATMs in epWAT of HFD-fed mice. (H) The frequencies of CD301(CLEC10A)-positive ATMs in overweight/obese and healthy weight individuals. ∗*P* < 0.05, ∗∗*P* < 0.01, ∗∗∗*P* < 0.001 mean the significant difference between groups, and ns means not significant. epWAT, epididymal adipose tissue.

CD14 is a marker expressed on monocytes and macrophages (8), which are essential components of the SVFs (26). To further investigate the role of the *Cd14*^−/−^ microenvironment in adipose angiogenesis, bEND.3 microvascular endothelial cells were conditioned with the supernatant of epWAT-harvested SVFs from either WT or *Cd14*^−/−^ mice and subsequently assayed for their tube formation potential. It was observed that bEND.3 cells formed more vascular-like structures when conditioned with the supernatant from *Cd14*^−/−^ SVFs compared to that from WT SVFs, regardless of whether the mice were fed a chow diet or a HFD (Fig. 2A). Moreover, *Cd14*^−/−^ SVFs-conditioned bEND.3 cells exhibited enhanced proliferation (Fig. 2B). These experiments prompted further investigation into the specific factors and types of ATMs involved in *Cd14*^−/−^-related adipose angiogenesis.

Macrophages are crucial immune cell types within AT, playing homeostatic roles both in steady-state conditions and during obesity. As obesity progresses, the phenotype and function of macrophages undergo significant alterations (27). In addition to their roles in inducing inflammation and insulin resistance, certain populations of ATMs display nonimmune functions, such as promoting angiogenesis through the secretion of proangiogenic factors (9, 28, 29). To explore the role of CD14 in this context, we compared the accumulation, phenotypic characteristics, and functional alterations of ATMs in epWATs from HFD-fed WT and *Cd14*^−/−^ mice. As shown in Fig. 2C, both *Cd14*^−/−^ and WT epWATs contained comparable proportions of total ATMs (F4/80^+^CD11b^+^). However, differentially expressed gene analysis revealed that *Cd301b* expression was significantly upregulated in *Cd14*^−/−^ epWATs (Fig. 2D), a finding that was corroborated by RT-qPCR results (Fig. 2E).

CD301b (macrophage galactose-type C-type lectin 2, *Mgl2*) is a lectin commonly utilized as a marker for alternatively activated macrophages. Previous studies have indicated that CD301b^+^ mononuclear phagocytes play crucial roles in maintaining glucose metabolism and overall energy balance (30). In subsequent experiments, it was observed that the frequency of CD301b^+^ cells increased in *Cd14*^−/−^ ATMs (Fig. 2F). Additionally, the loss of CD14 slightly reduced the proportion of antiinflammatory CD206^+^ ATM subsets without affecting proinflammatory CD11c^+^ subsets (Fig. 2G). This suggested that CD14 deficiency induced phenotypic shift in macrophages characterized by increased CD301b expression. In human overweight and obese individuals, who exhibited a pronounced increase in their proportion of CD14^+^ ATMs (Fig. 1H), CD301(CLEC10A), the ortholog of mouse CD301b was found to be decreased on CD68^+^CD11b^+^ ATMs (Fig. 2H).

These results suggested that CD14 deficiency had no effects on accumulation of macrophages in AT but facilitated their phenotypic adaptation into CD301b^+^ macrophages.

### CD14^low^CD301^+^ ATMs possess proangiogenic capacity

**Fig. 3:**
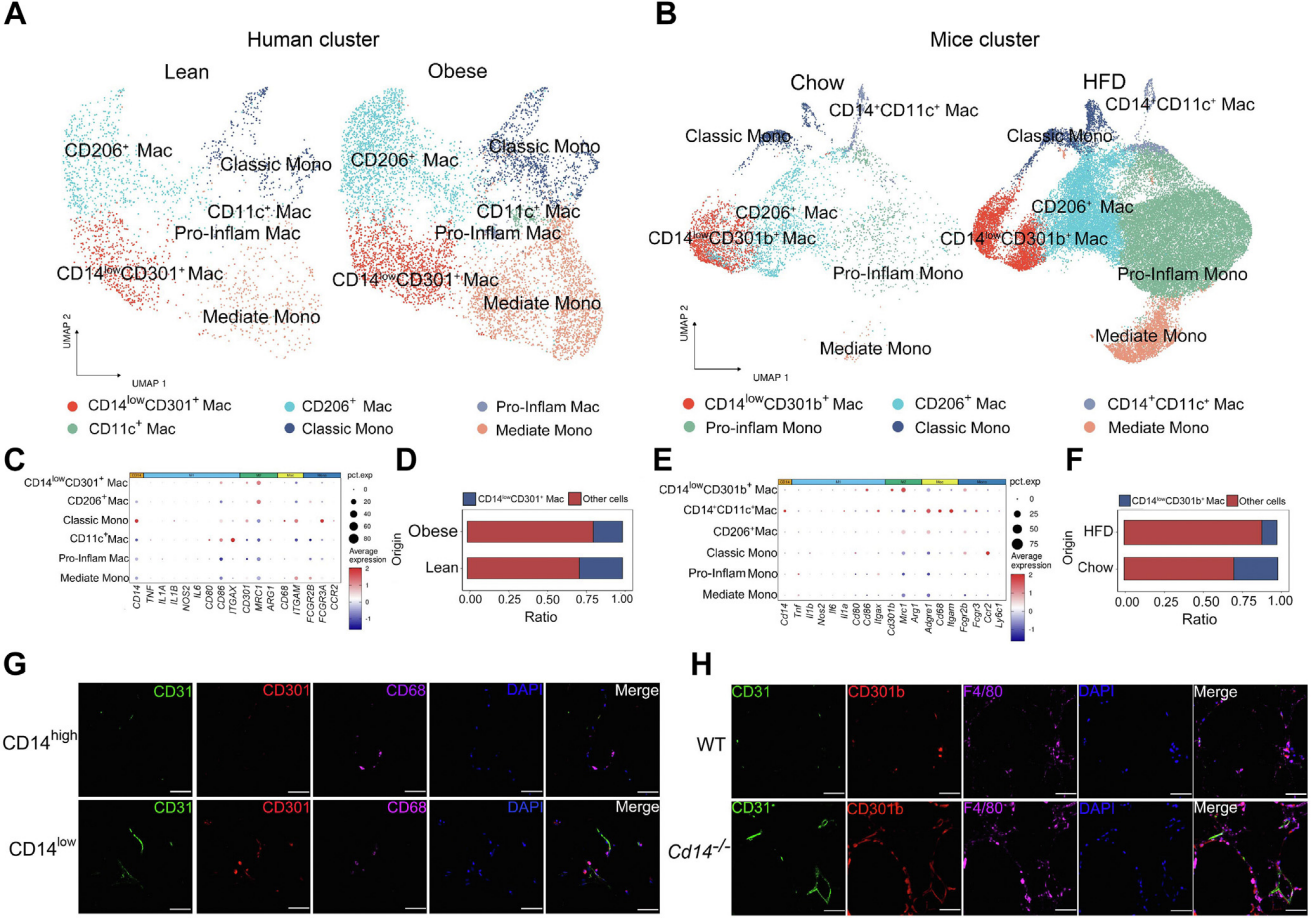
The proangiogenic potential of CD14^low^CD301^+^ ATMs. (A, B) UMPA presentation of six monocyte/macrophage clusters in (A) human and (B) mice adipose tissues. Clusters were annotated manually based on their respective protein and mRNA expression profiles. (C–F) Expression of marker genes in each Mono/Mac cluster and constitutive share of CD14^low^ CD301^+^ ATMs from (C, D) human and (E, F) mice. (G) Representative immunofluorescent images showed the expressions of CD31, CD301(Clec10a), CD68 in CD14^high^, and CD14^low^ human AT sections and (H) of CD31, CD301b, F4/80 in WT, and *Cd14*^*−/−*^ mice sections.

Despite numerous investigations into the various functions of macrophages in tissues, the specific distribution landscape of these cells in AT remains poorly understood. To elucidate the composition of monocyte-macrophages in AT and their functional distribution, a set of AT single-cell transcriptomic sequencing data (GSE176067) was analyzed. Following dimensionality reduction and clustering, we identified 16 distinct cell types, among which monocyte-macrophages were further clustered and identified into six cell clusters (Fig. 3A). These clusters were specifically designated as CD11c^+^ Mac, CD206^+^ Mac, CD14^low^ CD301(Clec10a)^+^ Mac, Mediate Mono, Classic Mono, and Pro-Inflam Mac, following the nomenclature established in several scSeq atlases. Notably, CD301-expressing macrophages exhibited lower CD14 expression compared to classic monocytes, and their marker gene expression profile differed from that of CD206^+^ M2-like Mac (Fig. 3C). Quantitative analysis revealed a significant decrease in the percentage of CD14^low^CD301^+^ macrophages in AT from the obese population (Fig. 3D), suggesting a negative correlation between overweight/obese states and the accumulation of CD14^low^CD301^+^ macrophages in human AT.

In mouse AT scRNA-seq, a similar cell composition with marker gene expression was observed. Consistent with the human data, CD301b-expressing macrophages in mice also demonstrated significantly lower CD14 expression (Fig.3B, E). Additionally, the frequency of CD14^low^CD301b^+^ macrophages in the monocytes-macrophages population of AT was significantly reduced in HFD-fed mice compared to chow-fed mice (Fig. 3F), reinforcing the negative association between obesity development and the CD14^low^CD301b^+^ macrophages accumulation in mice AT. The evidence suggests that CD301/CD301b-expressing macrophages may represent a distinct population of M2-like macrophages. Additionally, the expression of CD301/CD301b is negatively correlated with CD14 levels and the obesity development, highlighting the regulatory role of CD14^low^CD301^+^ macrophages in obesity progression. Furthermore, multicolor immunofluorescence analysis revealed that CD31 positive vessels were surrounded by CD68^+^CD301^+^macrophages in human AT (Fig. 3G) and by F4/80^+^CD301b^+^ macrophages in mice AT (Fig. 3H), indicating the proangiogenic potential of CD14^low^CD301^+^ ATMs.

### *Cd14*^*−/−*^ ATMs facilitate angiogenesis by secreting IGF-1

**Fig. 4:**
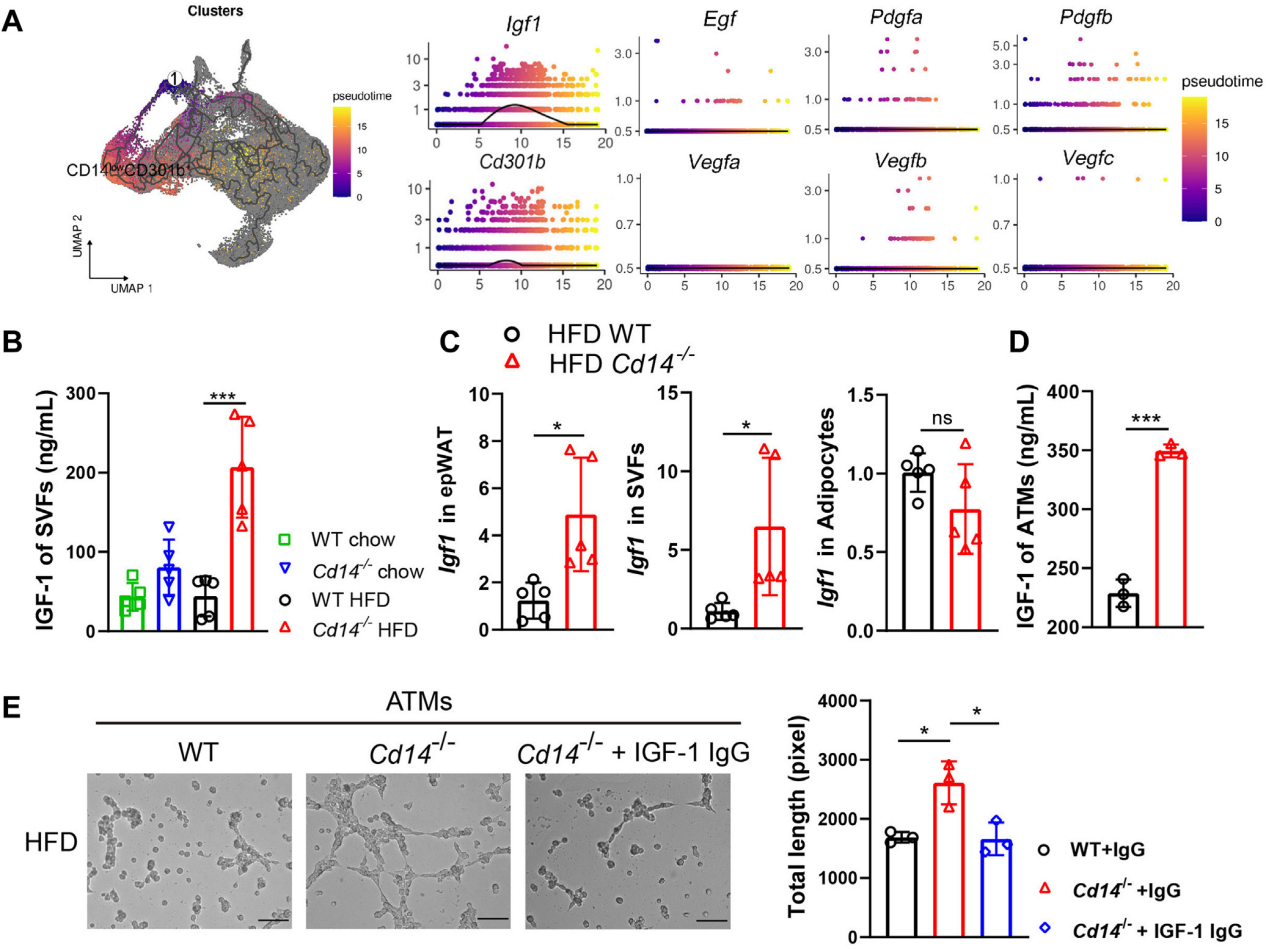
*Cd14*^*−/−*^ ATMs facilitate angiogenesis by secreting IGF-1. (A) Pseudotime analysis reveals differentiation of CD301b^+^ ATM in vivo. Left: UMAP plots depicting the inferred diffusion pseudotime of CD14^low^CD301b^high^ Mac in ATMs. Right: relative expression of *Cd301b*, *Igf1*, *Pdgf*, and *Vegf* across pseudotime. (B) ELISA assay for the concentration of IGF-1 released by SVFs. (C) RT-qPCR analysis for *Igf1* in epWAT (left), SVFs (middle), and adipocytes (right). (D) The concentration of IGF-1 in supernatants of WT and *Cd14*^*−/−*^ ATMs. (E) Tube formation by bEND.3 cells conditioned with ATMs supernatant. Scare bar: 100 μm. ∗*P* < 0.05, ∗∗∗*P* < 0.001 mean the significant difference between groups, and ns means not significant. epWAT, epididymal adipose tissue.

To elucidate the expression patterns of angiogenesis-related genes in CD14^low^CD301b^+^ ATMs, the expression levels of those genes were assessed in pseudotime. This analysis revealed that CD301b^+^ macrophages differentiated exclusively from classic monocytes. Notably, *Igf1*, which encodes IGF-1, was found to be elevated during the differentiation of CD14^low^CD301b^+^ macrophage, whereas *Vegfa*, *Pdgfa*, and other proangiogenic proteins did not show similar increases. Furthermore, the expression of *Igf1* was positively associated with pseudotime, as indicating by fitting additive models of *Cd301b* expression (Fig. 4A). This correlation indicated that the expression of both genes was interassociated. Consequently, *Cd14*^−/−^ SVFs were shown to secrete more IGF-1 than their WT counterparts following HFD challenge (Fig. 4B). Additionally, RT-qPCR analysis demonstrated that the loss of CD14 resulted in *Igf1* expression in epWAT, with this increment primarily originating from *Cd14*^−/−^ SVFs rather than adipocytes (Fig. 4C).

Since SVFs consist of various cell types, we aimed to clarify the source of IGF-1 by positively sorting ATMs from HFD-fed mice using F4/80^+^ magnetic beads and analyzing their IGF-1 secretion. The ELISA results indicated that *Cd14*^−/−^ ATMs, of which 61.12% ± 3.04% cells were CD301b positive as shown in Fig. 2F, produced significantly more IGF-1 compared to WT ATMs, where only 24.34% ± 1.75% were CD301b positive (Fig. 4D). Correspondingly, bEND.3 cells conditioned with supernatant from *Cd14*^*−*^^*/*^^*−*^ ATMs formed more vascular-like structures than those conditioned with WT ATMs. Notably, this proangiogenic effect was completely abrogated by the addition of IGF-1 IgG (Fig. 4E).

These data suggested that CD14 deletion enhanced the ability of ATMs to secrete more IGF-1, thereby promoting adipose angiogenesis.

### *Cd14*^−/−^ BMDMs promote angiogenesis via IGF-1

**Fig. 5:**
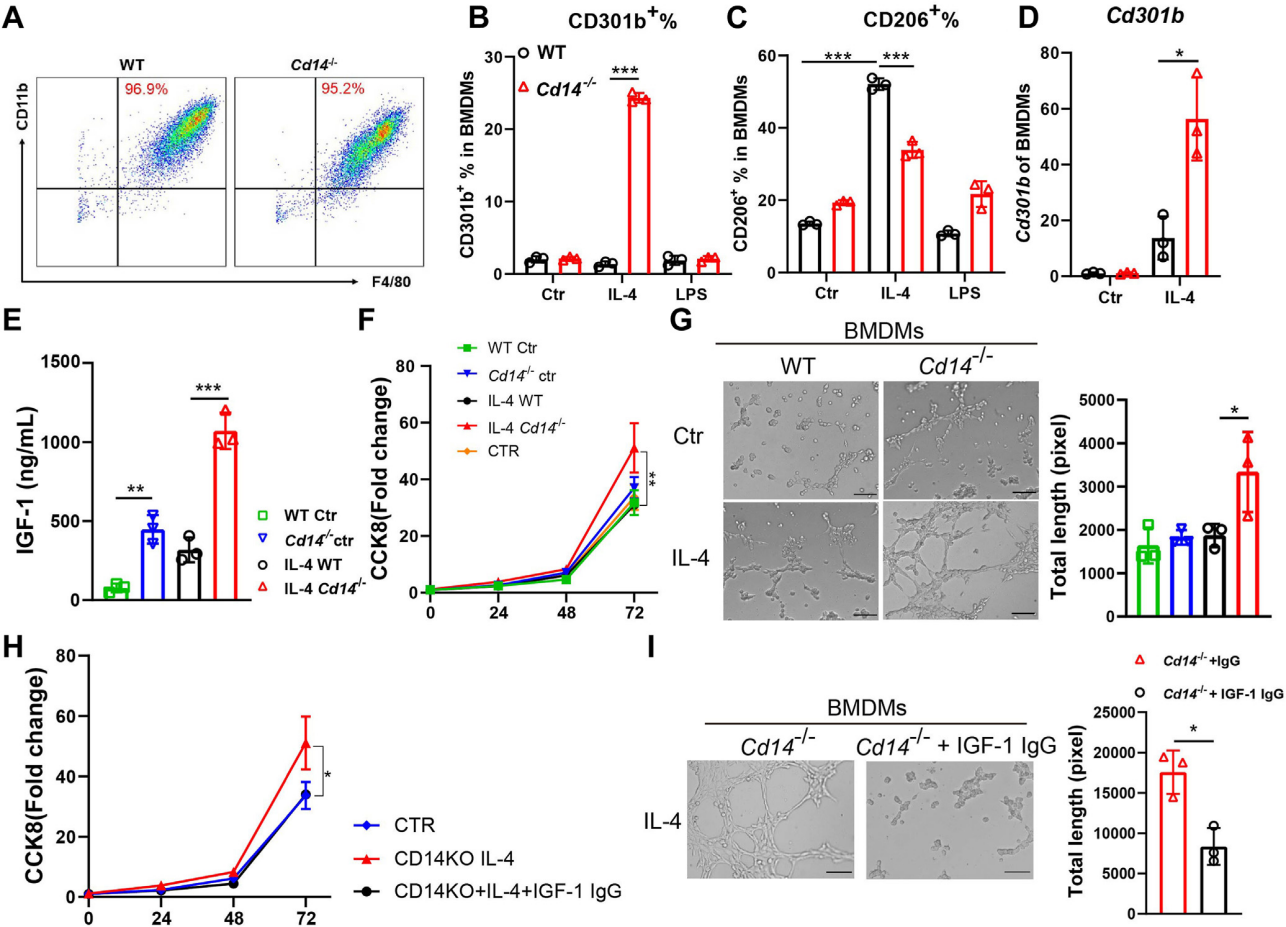
*Cd14*^−/−^ BMDMs promote angiogenesis via secreting IGF-1. (A) Induction of BMDMs. Frequencies of (B) CD301b^+^ and (C) CD206^+^ cells in IL-4 (20 ng/ml) or LPS (100 ng/ml) conditioned BMDMs. (D) *Cd301b* mRNA responsiveness to IL-4. (E) IGF-1 concentration in supernatants of BMDMs. (F) Proliferation of bEND.3 cells conditioned with BMDMs supernatants. (G) Tube formation by bEND.3 cells conditioned with BMDMs supernatants. (H) Proliferation and (I) tube formation with or without IGF-1 Abs blockade. Scare bar: 100 μm. ∗*P* < 0.05, ∗∗*P* < 0.01, ∗∗∗*P* < 0.001 mean the significant difference between groups, and ns means not significant. LPS, lipopolysaccharide.

To investigate whether *Cd14*^−/−^ BMDMs exhibit the same characteristics as *Cd14*^*−/−*^ ATMs, BMDMs were generated from 8-week-old mice and subjected to LPS or IL-4 induction. Notably, *Cd14* deﬁciency did not impact on macrophage generation (Fig. 5A), but it significantly increased the frequency of CD301b^+^ BMDMs and elevated *Cd301b* mRNA levels in BMDMs following IL-4 induction. In contrast, LPS induction did not influence CD301b expression. The other M2 macrophage marker, CD206, was expressed at a relatively low level in *Cd14*^−/−^ BMDMs compared to WT BMDMs upon IL-4 treatment (Fig. 5B–D). These findings indicate that both BMDMs and ATMs derived from *Cd14*^−/−^ mice exhibited a similar phenotype (Fig. 2F, G).

While bone marrow has been identiﬁed as a significant contributor to the ATM pool (31), BMDMs were utilized to assess whether *Cd14*^−/−^ macrophages exhibit proangiogenic properties. As illustrated in Fig. 5E, *Cd14*^−/−^ macrophages produced significantly higher levels of IGF-1 than WT cells (448.2 ± 89.8 ng/ml vs. 77.1 ± 27.4 ng/ml), and IL-4 treatment further amplified this difference (1,071.0 ± 114.7 ng/ml vs. 318.2 ± 78.7 ng/ml). Tube formation and cell proliferation analysis demonstrated that IL-4 treated *Cd14*^−/−^ BMDMs markedly enhanced bEND.3 proliferation and tube formation compared to their WT counterparts (Fig. 5F, G). However, this promotion was completely abolished by IGF-1 blockade (Fig. 5H, I). These findings confirmed that *Cd14*^−/−^ BMDMs also facilitated angiogenesis through IGF-1.

## Discussion

Numerous studies have established a positive association between CD14 expression and the development of obesity in human ATs (5, 6). Additionally, CD14 knockout mice have been reported to exhibit reduced lipid storage and improved insulin resistance, accompanied by decreased immune cell infiltration in both hepatic and ATs (7). In our research, we confirmed the positive correlation of CD14 with overweight and obesity in human subjects and observed that the loss of CD14 could alleviate HFD-induced obesity in mice. However, the precise mechanism by which CD14 regulated metabolic function remains unclear. To elucidate these underlying mechanisms, ATs from *Cd14*^−/−^ and WT mice were subjected to deep RNA-seq. Functional enrichment analysis indicated that CD14 was negatively correlated with angiogenesis-related function. Subsequent experiments validated the upregulation of CD31 in *Cd14*^−/−^ epWAT. These findings suggested that the relatively abundant sprouting vessels in *Cd14*^−/−^ ATs contributed to the amelioration of HFD-induced metabolic dysfunction. The enhanced angiogenesis observed in *Cd14*^−/−^ epWAT may explain why individuals with low CD14 expression exhibit a healthier body composition by decreased body fat, as improved angiogenic capacity is indicative of a beneficial pattern in AT expansion (32). AT is recognized as the most dynamic and plastic organ in adults, possessing the ability to expand or contract in response to varying metabolic challenges and nutrient status. Cocurrently, the blood vessel growth and regression occur. Angiogenesis is a critical factor for the healthy expansion of ATs and has been shown to play a significant role in the modulation of obesity (33, 34, 35, 36). Nevertheless, the influence of the CD14 microenvironment on angiogenesis within AT remains to be fully elucidated.

The analysis of differentially expressed genes provided valuable insights, suggesting that *Cd301b* was significantly upregulated in *Cd14*^−/−^ epWATs. CD301b is recognized as a marker of M2 macrophage, while CD14 is primarily expressed on the surfaces of monocytes and macrophages (3). Macrophages in AT have been reported to regulate adipose angiogenesis by secreting proangiogenic factors or expressing adhesion factors to support the proliferation and tube formation of endothelial cells (9, 28, 37). For example, LYVE-1^+^ ATMs have been shown to promote adipose angiogenesis, thereby preventing tissue hypoxia and enhancing metabolic communication between adipocytes and the circulation (14). Additionally, CD301b^+^ macrophages have been implicated in angiogenesis facilitated by calcium phosphate bioceramics (38) and are capable of secreting a variety of proangiogenic factors (38, 39, 40, 41). Moreover, CD301b^+^ macrophages exhibit potential in regulating metabolic functions, repairing damaged tissues, and stimulating vessel sprouting in diverse contexts (30, 38, 39, 42). In subsequent studies, we observed a significant increase in the frequency of CD301^+^ macrophages in *Cd14*^−/−^ mice that were protected from obesity and in healthy weight individuals with lower CD14 expression. Notably, these CD301^+^ macrophages were found to surround CD31^+^ endothelial cells. These findings confirm the negative correlation between CD301 and CD14 expression, suggesting that CD301b^+^ macrophages in *Cd14*^−/−^ epWAT may ameliorate dysfunction of ATs by enhancing the impaired vascular remodeling. Based on these results, it can be speculated that the aggregation of CD14^lo^CD301^+^ macrophages may serve as a proangiogenic marker in ATs.

The regulation of angiogenesis in AT by CD301b^+^ macrophages in *Cd14*^−/−^ microenvironment remains both obscure and intriguing. CD301b^+^ macrophages have been reported to secrete proangiogenic factors, including IL-27, IGF-1, Relmα, and VEGFa, across different disease models and tissues (38, 39, 40, 41). Among these factors, IGF-1 has been identified as being associated with pseudotime through the application of additive models on CD301b^+^ macrophages in ATs. IGF-1 plays a critical role in stimulating angiogenesis, particularly by promoting the tube formation, proliferation, and migration of endothelial cells (43). In this study, *Cd14*^−/−^ ATMs, SVFs, and *Cd14*^−/−^ BMDMs all exhibited increased IGF-1 production. Given that these cell populations contained elevated levels of CD301b^+^ macrophage, it can be speculated that the expression of CD301b is positively correlated with IGF-1production. Tube formation assays confirmed that IGF-1 was the primary proangiogenic factor secreted by *Cd14*^−/−^ macrophages, as blockade of IGF-1 completely abrogated the angiogenic enhancement observed. It is reasonable to hypothesize that by inhibiting CD14, various factors within the complex AT microenvironment regulate IGF-1 production by CD301b^+^ ATMs.

Previous findings have reported that CD301b^+^ macrophages express various proangiogenic factors, including VEGF, PDGF, and IGF-1(38, 42). However, in this study, only IGF-1 is observed to be upregulated in CD14^low^CD301b^+^ ATMs. A possible explanation for this discrepancy is that the function and differentiation of macrophages are influenced by distinct tissue microenvironments. For instance, CD301b^+^ macrophages secrete more VEGFa during bone regeneration (38) but release higher levels of IL-27 in the skin wound healing process (40). The microenvironment in ATs is markedly different from that of bone and skin. Various factors within the ATs microenvironment, including adipokines, metabolites, and proinflammatory or antiinflammatory cytokines, can affect the status of ATMs and subsequently alter the secretion profile of CD301b^+^ macrophages in ATs. This phenomenon warrants further investigation.

In this article, we elucidated the connection between CD14 and metabolic regulation through its impact on angiogenesis with AT. Several alternative hypotheses are proposed. For instance, intestinal resident macrophages lack the expression of CD14, resulting in a diminished production of proinflammatory cytokines against commensal bacteria (44). Consequently, CD14 may affect the composition of the intestinal ﬂora, which in turn could impact food absorption and weight gain. Another consideration is that CD14, functioning as a coreceptor with TLR4 (3, 8), may synergistically bind with LPS to promote obesity, as LPS levels alone are sufﬁcient to predispose individuals to obesity, insulin resistance, and type-2 diabetes (45). Additionally, the potential for CD14-expressing ATMs to perform no-immune functions, such as regulating thermogenesis and phagocytosing metabolites (9), warrants further investigation.

In conclusion, here we report that a CD14-deficient microenvironment in AT promotes the accumulation of CD301^+^ macrophages and enhances IGF-1 production, thereby facilitating adipose angiogenesis. The increased vascular density alleviates adipocyte hypoxia, which in turn reduces inflammation within ATs. This process supports healthy AT expansion and improves metabolic communication between adipocytes and the circulatory system, ultimately contributing to body composition regulation by decreasing body fat. These findings provide significant and novel insights into the role of CD14 in regulating angiogenesis in ATs and intricate relationship between CD14 expression and the development of obesity.

## Data availability

All data generated and analyzed in this manuscript are available from the corresponding author upon request.

## Supplemental data

This article contains supplemental data.

## Conflicts of interest

All authors have no relevant conflicts.
