# Supplementary material for: CD14loCD301b+ macrophages gathering as a proangiogenic marker in adipose tissues
Source: J Lipid Res. 2024 Dec 5;66(1):100720. doi: 10.1016/j.jlr.2024.100720 (PMC11745947; doi:10.1016/j.jlr.2024.100720)
Supplement: Supplementary materials [file mmc1.docx]

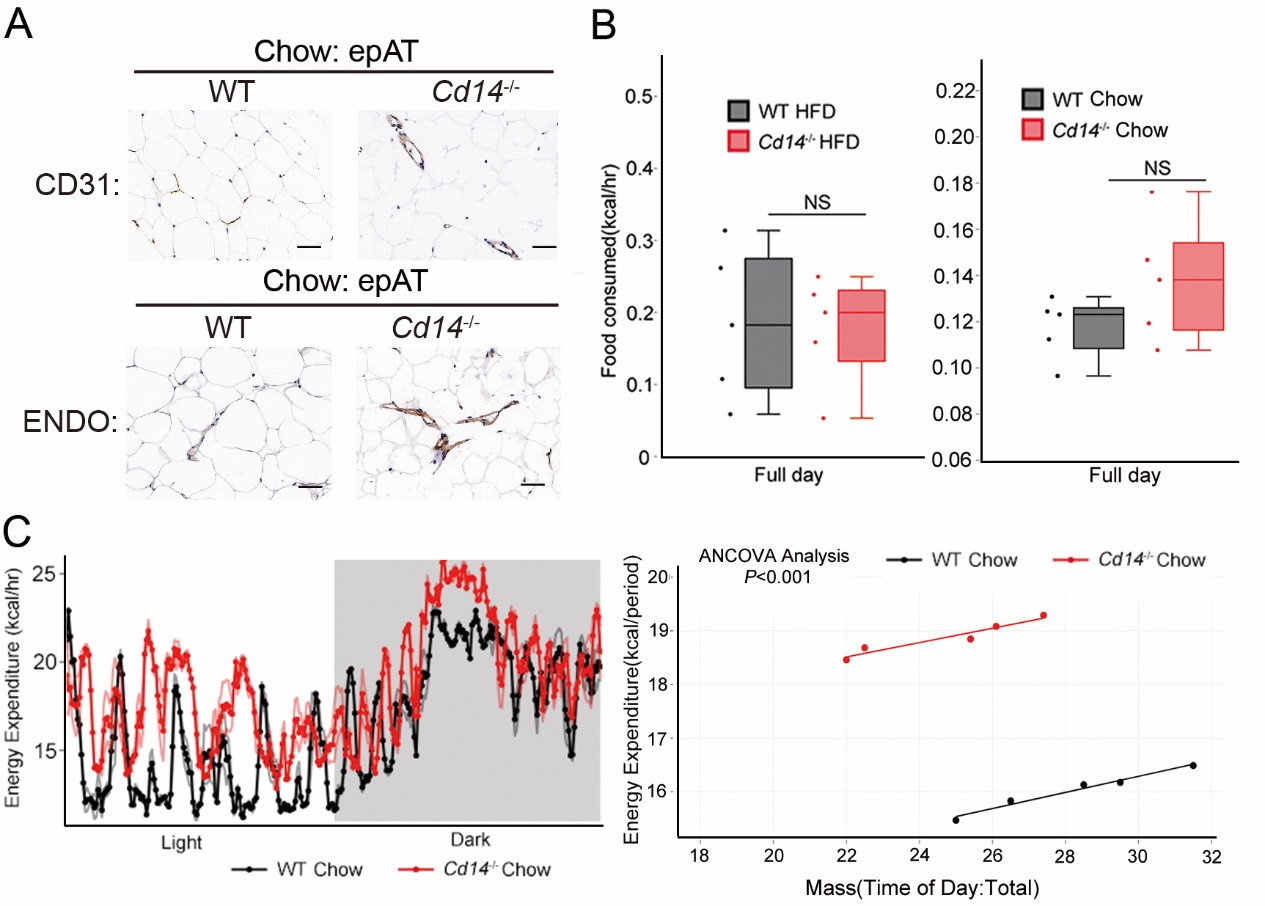


Supplementary Figure1: (A) Immunohistochemical staining for CD31 (left) and Endomucin (right) in epWATs of Chow-fed WT and *Cd14*^-/-^ mice (Scare bar: 50 μm). (B) Food intake of HFD-fed (left) or Chow-fed (right) WT and *Cd14*^-/-^ mice. (C) Energy expenditure curves (left) and ANCOVA analysis (right) of Chow-fed (right) WT and *Cd14*^-/-^ mice.

**Primer sequence:**

**Mouse:**

*Cd31:*

Forward ACGCTGGTGCTCTATGCAAG

Reverse TCAGTTGCTGCCCATTCATCA

| *Mgl2:*   \| Forward \| TTAGCCAATGTGCTTAGCTGG \| \| --- \| --- \| \| Reverse \| GGCCTCCAATTCTTGAAACCT \|   *Igf1:*   \| Forward \| CTGGACCAGAGACCCTTTGC \| \| --- \| --- \| \| Reverse \| GGACGGGGACTTCTGAGTCTT \|   **Human:**  *CD31:*  Forward AACAGTGTTGACATGAAGAGCC  Reverse TGTAAAACAGCACGTCATCCTT |  |
| --- | --- | --- | --- | --- | --- | --- | --- | --- | --- |
